# Oral Chagas Disease in Colombia—Confirmed and Suspected Routes of Transmission

**DOI:** 10.3390/tropicalmed9010014

**Published:** 2024-01-04

**Authors:** Norman L. Beatty, Catalina Arango-Ferreira, Lídia Gual-Gonzalez, Sara Zuluaga, Melissa S. Nolan, Omar Cantillo-Barraza

**Affiliations:** 1 Division of Infectious Diseases and Global Medicine, Department of Medicine, University of Florida College of Medicine, Gainesville, FL 32610, USA; norman.beatty@medicine.ufl.edu; 2 Emerging Pathogens Institute, University of Florida, Gainesville, FL 32610, USA; 3 Departamento de Pediatría, Hospital San Vicente Fundación, Medellín 050010, Colombia; catalina.arango@sanvicentefundacion.com; 4 Departamento de Pediatría, Facultad de Medicina, Universidad de Antioquia, Medellín 050010, Colombia; 5 Arnold School of Public Health, University of South Carolina, Columbia, SC 29208, USA; lidiag@email.sc.edu (L.G.-G.); msnolan@mailbox.sc.edu (M.S.N.); 6 Grupo Biología y Control de Enfermedades Infecciosas, Universidad de Antioquia, Medellín 050010, Colombia; sara.zuluagaa@udea.edu.co

**Keywords:** Chagas disease, oral transmission, vector-borne zoonosis, *Trypanosoma cruzi*, *Panstrongylus geniculatus*, armadillo blood, *Didelphis marsupialis*

## Abstract

Chagas disease (CD) remains endemic throughout many regions of Colombia despite implementing decades of vector control strategies in several departments. Some regions have had a significant decrease in vectorial transmission, but the oral ingestion of *Trypanosoma cruzi* through consumption of contaminated food and drink products is increasingly described. This form of transmission has important public health relevance in Colombia due to an increase in reported acute CD cases and clinical manifestations that often lead to significant morbidity and mortality. Oral CD in Colombia has been associated with the consumption of contaminated fruit juices, such as palm wine, sugar cane, or tangerine juice and water for consumption, or contaminated surfaces where food has been prepared. Another interesting route of oral transmission includes ingestion of unbeknownst infected armadillos’ blood, which is related to a traditional medicine practice in Colombia. Some earlier reports have also implemented consumption of infected bush meat as a source, but this is still being debated. Within the Amazon Basin, oral transmission is now considered the principal cause of acute CD in these regions. Furthermore, new cases of acute CD are now being seen in departments where CD has not been documented, and triatomine vectors are not naturally found, thus raising suspicion for oral transmission. The oral CD could also be considered a food-borne zoonosis, and odoriferous didelphid secretions have been implemented in contaminating the human dwelling environment, increasing the risk of consumption of infectious metacyclic trypomastigotes. In this article, we will discuss the complex transmission dynamics of oral CD in Colombia and further examine the unique clinical manifestations of this route of infection. New insights into the oral transmission of *Trypanosoma cruzi* are being discovered in Colombia, which can help bring increased awareness and a better understanding of this neglected tropical disease to reduce the burden of CD throughout Latin America.

## 1. Introduction

**Figure 1:**
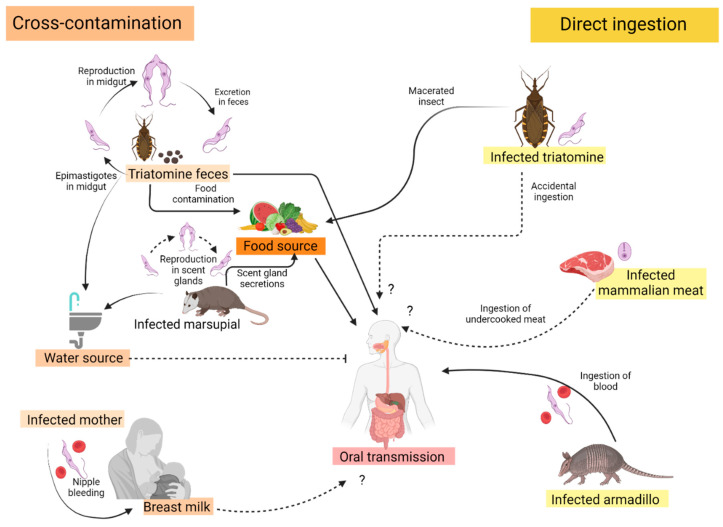
Confirmed (black arrow) and proposed (dotted black arrow) sources of oral transmission of Chagas disease in Colombia.

Oral transmission of the protozoan parasite *Trypanosoma cruzi* is one mode of acquisition that can occur among those living or traveling to endemic regions. Increasing awareness of oral transmission is occurring, and some regions are now showing increased frequency via the oral route [1]. Concerns for oral transmission of *T. cruzi* were first mentioned by Carlos Chagas and then experientially confirmed in 1921 with the oral consumption of blood trypomastigotes and then with triatomine feces in 1933 [2]. Since that time, the oral ingestion of contaminated food and/or drinks that contain the parasite, or even undercooked or raw, infected mammalian bush meat, has been demonstrated or speculated to cause CD [3] (Figure 1). Another potential source of contaminated environmental exposure is the odoriferous didelphid secretions from the anal gland of an infected marsupial such as *Didelphis marsupialis* [3,4]. The parasite naturally circulates between the triatomine insect vector (more commonly known as the “pito” in Colombia) and other susceptible sylvatic, peridomestic, and domestic mammals and potential reservoirs (including canines and humans) to complete its life cycle [4]. Oral transmission routes in sylvatic cycles occur in two distinct ways. This includes uninfected mammals consuming and eating an infected mammal and uninfected mammals consuming an infected triatomine insect. Throughout Colombia, vectorial transmission persists in several regions like the municipalities of Aguachica, Yopal and Paz de Ariporo (Casanare), Sierra Nevada de Santa Martha, and in the departments of Magdalena, La Guajira, Boyacá, Santander, Bolivar, Arauca, Antioquia, Chocó and Cesar, although data are limited for most of Colombia [5,6,7]. Campaigns targeting vector control over several decades (Iniciativa de Países Andinos) have been successful at reducing intradomestic vectorial transmission caused by *Rhodnius prolixus* [7,8]. Reducing or eliminating the presence of *R. prolixus* in and around human dwellings has led to other triatomine species filling that void in the food web. This includes the triatomine species, *T. dimidiata*, *T. venosa*, *T. maculata*, *and R. pallescens*. These largely sylvatic and peridomestic triatomines are now being shown to commonly invade human inhabitants and raise concern for emerging CD vectors in Colombia [9,10].

**Figure 2:**
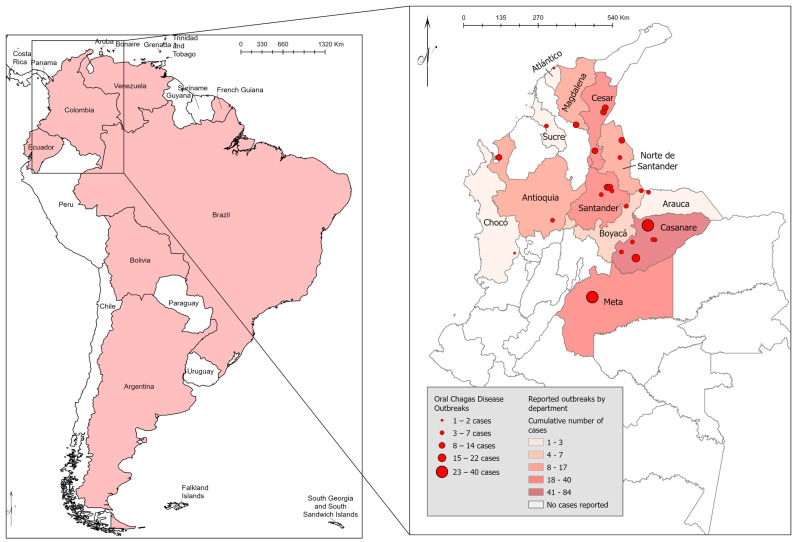
Countries in South America (**left**) where oral Chagas disease has been described and cumulative cases as documented in Colombia (**right**) where outbreaks have been reported to Instituto Nacional De Salud (INS).

The countries in South America where oral transmission of *T. cruzi* to humans has been described include, Argentina, Bolivia, Brazil, Colombia, Ecuador, French Guiana, and Venezuela [2,3,4] (Figure 2). The first well-described case of oral CD in 1965 was reported in Brazil [11]. In Colombia, suspected or confirmed oral CD cases or outbreaks are registered with the Instituto Nacional de Salud (INS). These cases are often described in the Boletín Epidemiológico Semanal (BES), among other published reports (Table 1). Chagas disease endemic regions, such as those found in Mexico and regions of Central America, have limited or no information on oral transmission, but it is likely occurring. Overall, among the countries with known oral transmissions, various isolated single case reports among an individual or family who consumed contaminated food or drink are reported [3,4]. However, large outbreaks have also been described where dozens of people were known to acquire the infection from one contaminated product [11,12,13,14]. Distinct from vectorial transmission, *T. cruzi* orally consumed is mediated by gp82 and gp90 parasite glycoproteins [15]. Parasite discrete typing units (DTUs) are associated with variance in glycoprotein expression, which influences their capacity to adhere to and enter gastric epithelial cells [16]. Colombia has now begun to see increased recognition of oral transmission of CD, which is likely due to the clinical significance of those infected in this manner. For those who acquire the infection via the oral route, it is common to manifest signs and symptoms of acute CD [17,18]. For other forms of CD, like the vector-borne infection, this is uncommon, and most will either be asymptomatic or only develop a mild, self-resolving febrile illness [1]. Given the overall tremendous load of *T. cruzi* ingested from oral exposure, most individuals will have significant clinical disease, and mortality can reach as high as 35% [1].

In this review article, we will discuss the many facets of oral transmission of *T. cruzi*. This will include the ingestion of contaminated drinks such as fruit juices and the consumption of infected mammalian bush meat. We will review other unique routes of oral consumption of the parasite, including ingestion of mammalian blood among certain cultural practices in Colombia. We will review the pathophysiology surrounding this route and the clinical differences seen among those infected via oral transmission versus those with other forms, such as vectorial. Our team will also provide some new insights into risks for oral transmission in Colombia, including infected triatomine feces as environmental contamination with certain peridomestic species such as *Panstrongylus geniculatus*. Lastly, we will review the increasing concern that odoriferous didelphid secretions can serve as an alternative source of oral transmission of *T. cruzi* through contamination of the environment with these infected secretions.

## 2. Clinical Manifestations of Oral Chagas Disease

Since the first reported evidence of oral transmission in 1965, outbreaks of oral Chagas disease have gained importance as an emerging route of transmission. In some regions, it is considered to be the transmission mode in up to 50% of cases in certain geographical locations like in the Amazon basin [40]. Due to the severity of the disease, many who become infected via oral ingestion will manifest significant signs and symptoms of acute CD, which can lead to fulminant myocarditis and heart failure, meningoencephalitis, and even life-threatening shock from parasitemia [11,12,13,14,17,18,40,41].

The incubation period following the oral ingestion of *T. cruzi*-contaminated products is approximately 3–22 days, in contrast to 4–15 days for vectorial transmission and 8–160 days for transfusion- and transplant-related transmission [1]. A shorter incubation period is likely due to the overall significantly increased parasite load compared to the other routes of transmission. Symptoms and rapid disease progression in those who are immunocompetent are not common among other forms of transmission like vectorial, congenital, or transfusion-related transmission [4]. The vast majority of those with acute oral Chagas have fever (71–100%), but other systemic symptoms are notable and include facial edema, lower extremity edema, myalgia, generalized lymphadenopathy, abdominal discomfort, dyspnea, vomiting, diarrhea, hepatomegaly, splenomegaly, headache, chest pain, cutaneous erythematous rash, jaundice, arthralgia, epistaxis, hematemesis, melena, and palpitations [11,12,13,14,17,18,40,41]. Facial edema, typically involving the entire face and portions of the lips, is present in 57–100% of those with acute oral CD [4,40]. This can be differentiated from vectorial transmission, where we more commonly see unilateral periorbital swelling (a.k.a. Romaña’s sign) in those with acute symptoms [4,40]. The robust systemic immune response that is seen in those with acute oral CD is suggested to occur due to a more efficient transmission after penetration through the oral, pharyngeal, and gastric mucosa. In addition, we also see many-fold higher parasitic loads present in contaminated food and drink products as compared to vectorial transmission, and thus exacerbated clinical signs and symptoms of infection. It has been estimated that a single crushed triatomine harboring *T. cruzi* can contain 600,000 metacyclic trypomastigotes as compared to 3000 to 4000 per microliter of infected triatomine fecal material [4].

The largest outbreak of orally transmitted Chagas was linked to contaminated guava juice consumption in a Venezuelan elementary school located in Caracas [13]. The outbreak reported a total of 119 confirmed and suspected cases of CD. Clinical courses of those confirmed or suspected cases reported 75% were symptomatic and 20.3% required hospitalization. One five-year-old child died of acute myocarditis [13]. These percentages differ from those reported in vectorial transmitted Chagas, reported to be asymptomatic for the acute phase of infection in up to 95–99% of cases [1]

Cardiac abnormalities are seen more frequently after oral transmission of *T. cruzi* as opposed to vectorial transmission [40,41,42,43]. Of these, electrocardiographic abnormalities are reported in a majority of patients, specifically ventricular polarization disturbances and pericardical involvement in young soldiers in Colombia [40,42]. Electrocardiographic abnormalities in oral Chagas, as described in the largest reported outbreak (N = 103), were present in 66% of confirmed cases and are reported with a predominance in children under 18 years old compared to adults (69.7% vs. 56%) who were infected [42]. The more frequently observed electrocardiogram (ECG) alterations include ST segment and T wave abnormalities (37%) as well as QT prolongation (2.9%) [42]. The right bundle branch block, which is common in chronic Chagas heart disease (5–25% of chronic Chagas heart disease), was also seen in acute oral CD but with a much lower frequency at 1.94% (N = 2/103), and the left bundle branch block was seen at 2.9% (N = 3/103) [42]. ST segment and T wave abnormalities were seen between both age groups and were more common in <18 years (72% versus 19%) as opposed to adults [42]. Echocardiograms in the mentioned study were performed if the ECG was abnormal (66%; N = 68/103). This revealed 32% (N = 22) with mild to moderate pericardial effusion seen on echocardiogram as well as 33% (N = 33/103) with arrhythmias. This includes 22% (N = 23/103) supraventricular arrhythmias, ventricular arrhythmias 5.8% (N = 6/103), and atrioventricular block 2.9% (N = 3/102) [42]. Ventricular dysfunction with low ejection fraction has also been described in 27% of cases of oral CD [41].

## 3. Oral *T. cruzi* Transmission Pathogenicity

*Trypanosoma cruzi* is a generalist parasite that can infect >136 triatomine vector species, essentially any mammal, and almost all mammalian tissues [44,45]. A flagellate kinetoplast, this parasite’s life cycle involves three distinct forms, with the trypomastigote and amastigote forms of clinical relevance. The traditional *T. cruzi* pathogenesis follows the route of trypomastigote systemic circulation, adherence to smooth muscle tissue, conversion to the amastigote form, intracellular amastigote reproduction and nest expansion, eventual cellular damage yielding parasite egress, and the cycle repeats with newly formed trypomastigotes systemically circulating [46]. *T. cruzi* demonstrates tropism for cardiac and gastrointestinal smooth muscle tissues, although the parasite can be found disseminated throughout the human body [45,47]. Intracellular parasite growth produces a pronounced inflammatory infiltrate response, with cellular damage presenting as inflammatory lesions and fibrosis [46]. The inflammatory infiltrate can directly destroy neurons and cardiac fibers, in contrast to the physical fibrotic damage caused by necrotic amastigote nest hollows [47].

This cycle slows down and becomes subclinical during the intermediate phase, which lasts indefinitely without chemotherapeutics. In approximately 30% of patients, this cellular damage cycle cryptically continues at an accelerated pace until advanced disease clinically manifests. The factors that contribute to pathogenesis and reactivation are largely unknown but are hypothesized to correlate with parasite discrete typing unit [48,49,50], high-fat diets [51,52], co-infections [53,54], and host immunogenetics [55]. Murine models indicate three potential pathogenic pathways collectively, independently, or in verse contribute to cellular damage: autoimmune, neurogenic, or pro-inflammatory damage mediated by certain cytokines such as interleukin (IL)-6 and IL-17 [3,46,56,57,58,59,60,61]. While evidence supports all three pathways, the strongest contemporary evidence supports a hybrid pathogenic mechanism of host autoimmune activation and persisting parasitic material that stimulates inflammatory host response [60].

Most people who have been infected with the parasite are thought to have acquired *T. cruzi* via the vector-borne route. This involves an infected triatomine defecating fecal material containing metacyclic trypomastigotes on an individual while being bitten. The parasite can then enter the body through the mucosa or a breach in the skin at or near the bite site. The rest of those who acquire the infection may do so via oral consumption, pregnancy, or other avenues. Given the recent emergence and rarity of oral *T. cruzi* transmission, the pathophysiology scientific literature is limited. Case reports verify that this mechanism is associated with greater disease manifestations and high mortality, suggesting an alternative acute disease pathogenesis process occurs. More research is needed to better understand this neglected route of *T. cruzi* infection.

## 4. *T. cruzi* Contaminated Food and Drink

### 4.1. Ingestion of T. cruzi Contaminated Fruit Juices and Foods

Oral CD in Colombia has been associated with the possible consumption of contaminated fruit juices and food. In other mammalian hosts, the oral way is the most important transmission form [62]. In humans, it is debated which are the main sources of oral infection, from contaminated food through triatomine feces or directly macerated triatomines in beverages and fruit juices [3]. These contaminated food sources combine specific aliments that are more likely to be contaminated by insects or insect feces; however, any food or drink left unattended could potentially become contaminated from possible animal scent gland secretions [14,63]. Most of the time, the source of these oral transmissions is not described; however, there are two beverages in Colombia that have been associated with oral CD: palm wine and tangerine juice [3,26,27]. In 1999, during an outbreak in Guamal (Magdalena) municipality, 18 cases were found associated with palm wine (“vino de palma”), a fermented drink common in some Colombian regions [20]. This drink is prepared by performing a deep cut in the palm to reach the heart to obtain the sap, collecting several liters that are left to ferment. Sometimes, this drink is consumed immediately, and it could be infected by triatomine feces, which could then transmit the parasite orally. An outbreak in 2008 was associated with tangerine juice and was described as a source of orally acquired CD in Santander. It affected nine individuals who visited the same farm, and all had the same juice for breakfast [21,23]. The possible source was thought to be orange juice contaminated with insect feces contaminating the oranges which made the juice, or infected triatomines macerated within the sugar cane, that would have been posteriorly mixed with the juice and infecting the individuals consuming that juice [13,64]. Since orange juice is not macerated but squeezed, it is not clear how the juice becomes contaminated, and thus, the speculation on sugar cane contamination. A case series published in 2022 described two pediatric cases of acute oral Chagas disease, supposedly related to the consumption of sugar cane juice, also called “guarapo de caña” in Spanish [17]. Similarly, animal studies demonstrate sugar cane juice can remain infective for up to twenty-four hours post-inoculation [64].

Açai juice is a common source of orally acquired Chagas disease in Brazil, although Colombians have some national açai fruit farms and regularly import this juice from Brazil. It is thought the infected triatomines or triatomine feces-contaminated fruits are macerated with the fruits, thus contaminating the drink [62]. In an experimental study, they observed the parasite to survive between 24 and 72 h in several drinks, including mandarin, guayaba, and guanabana, and up to 384 h in guanabana when preserved at 4 °C; results were reproduced in a separate study with similar conditions for açai juice [65,66]. Further, some populations culturally use açai juice as a way to wean infants off breastmilk, and infants have developed Chagas cardiomyopathy following consumption of contaminated açai juice [67]. Considering juices are usually consumed within a short amount of time after preparation, it is important to note how 24 h would make this transmission route feasible and the most likely. No other juices and foods have been directly documented to be the source of infection in Colombia, but other ways of contamination of food and beverages can happen and should be considered.

### 4.2. Consumption of T. cruzi Infected Mammalian Meat

When considering CD as food-borne, we need to look at the parasite’s life cycle. In the *T. cruzi* life cycle, the trypomastigotes in the blood will turn into an amastigote inside the muscle cells of the infected animals and humans [68]. It is discussed whether the infected muscle cells can be a source of infection in humans when eating infected mammalian meat. It has been demonstrated experimentally that opossums (*Didelphis albiventris*) can become infected by feeding on mice infected with *T. cruzi*, thus bringing attention to the possibility of oral transmission in humans through consumption of undercooked or raw infected meat [62]. In 2016, Sangensis et al. performed a systematic review evaluating the transmission of *T. cruzi* through the consumption of game meat, such as the nine-banded armadillo (*Dasypus novemcinctus*) [69]. The transmission through infected meat is considered rare, though possible, and at the same time has brought some controversies on whether there might have been infection through manipulation of the animal carcass during the butchering of the animal and the cooking process that led to cross-contamination and later infection [70]. In Colombia, the consumption of possibly infected wild animals such as armadillo (*Dasypus* species), opossum (*Didelphis* species), and lowland paca (*Cuniculus paca*) is common, regardless of being reservoirs for zoonotic diseases, and the butchering process is usually performed under unhygienic conditions that could potentially expose those manipulating animals and causing cross-contamination of the instruments used for cooking [24,71,72,73]. Although there is a lack of evidence of transmission of *T. cruzi* from infected mammalian meat to humans, lowland paca (also known as “lapa”, “guagua” o “guartinaja”) has been associated with *T. cruzi* infection vectored by *P. geniculatus*. This bush meat is one of the most appreciated meats in the country, thus, infection through this route should be considered and evaluated [74]. Subsistence hunters among indigenous and rural communities within the southern Colombian Amazon, including the departments of Amazonas, Putumayo, Caquetá, Guainía, Guaviare, Vaupés, and other sections of Cauca, Meta, and Vichada, likely consume other known *T. cruzi* infected mammals, including the *Nasua nasua* or South American coati, the “kinkajou” (*Potas flavus*) and non-human primates [75,76]. Risks for consuming un- or undercooked *T. cruzi*-infected mammalian bush meat are not well understood among this population and could be considered a source of oral ingestion of the parasite.

### 4.3. Ingestion of Infected Mammalian Blood

In addition to the “traditional” food-borne transmission route by manipulating contaminated carcasses and contaminated food sources, some traditional cultural practices relate to the oral *T. cruzi* transmission. An example of those in Colombia and other Latin American countries is the use of armadillo blood (*D. novemcinctus*) and other body parts for their believed medicinal properties [71,77]. Nine-banded armadillos, called “gurre” in the Andean region of Colombia, are well-distributed wildlife in Colombia, and these are insectivorous mammals and can become *T. cruzi* reservoirs from ingestion of triatomine bugs, potentially becoming a source of infection [14]. Besides being used as a meat source, armadillos are captured and used for their supposed medical properties: the tail and shell are turned into a powder to relieve adverse effects of pregnancies, its fat is used to cure inflammation and ear pain, or varicose veins, and the blood is drunk to relieve asthma symptoms and other respiratory conditions [78,79,80,81]. This cultural practice can bring undesired infections, especially the consumption of infected blood and undercooked meat [82,83]. In 2019, in Chocó, San José del Palmar, two family members were diagnosed with *T. cruzi* infection within a short period of time after both cases had allegedly consumed armadillo blood and, thus, obtained the infection from the consumption of such blood [31]. As a traditional cultural practice, it is important to consider this infection route to develop targeted interventions that are culturally sensitive to prevent additional cases.

### 4.4. Other Unique Forms of T. cruzi Oral Ingestion

Oral CD transmission is most recognizable when a cluster of acute CD cases is seen, presenting with clinical manifestations which is less likely to be seen in vector-borne transmission [4]. Accidental bug ingestion has been considered a possibility of oral transmission, which can occur if a triatomine becomes macerated within the food or beverages being prepared [4,24,79,80]. The possibility of infection through ingestion of the bug relies on the triatomine parasite load, which can vary between species and the timing of previous infectious blood meal [84,85,86]. Although it is difficult to assemble evidence of an orally acquired infection after ingesting an infected triatomine insect, it is a plausible route of oral *T. cruzi* infection in humans.

Some other forms of ingestion have been discussed in the literature. It has been considered that acutely infected mothers who were breastfeeding could potentially transmit the parasite orally from their breast milk [87]. Trypomastigotes have been found in the breast milk of several mothers that were in the acute and chronic phase through direct examination of the parasite; however, only one has been seen positive using xenodiagnoses, suggesting it to be unlikely to transmit the infection orally [88,89,90]. Some authors suggest that breast milk is a poor mechanism of transmission and that breast milk could potentially become cross-contaminated in mothers who have bleeding nipples and not from the milk itself [88]. Although it seems unlikely, in experimental settings, successful transmission of *T. cruzi* using unpasteurized human milk has been observed in mice [91]. However, no clinical evidence supports oral transmission naturally through breast milk, and further research should be conducted to consider this source of oral transmission.

Water contamination has been mentioned as a source of *T. cruzi* infection [24,62]. Referencing an outbreak in Brazil, where the source of infection was suggested to be inadequately stored soft drinks and/or water [92]. The authors did not confirm water as the source of infection; thus, the suggestion has been considered not plausible, from experimental studies where the parasite does not show survival in water, eliminating the possibility of water being a source of infection [65,93]. Given the lack of evidence supporting water as the source of infection, it is likely that it is not a good transmission mechanism, and epidemiological studies should probably not consider it as the source of infection.

## 5. Peridomestic Triatomines Associated with Oral *T. cruzi* Transmission

**Figure 3:**
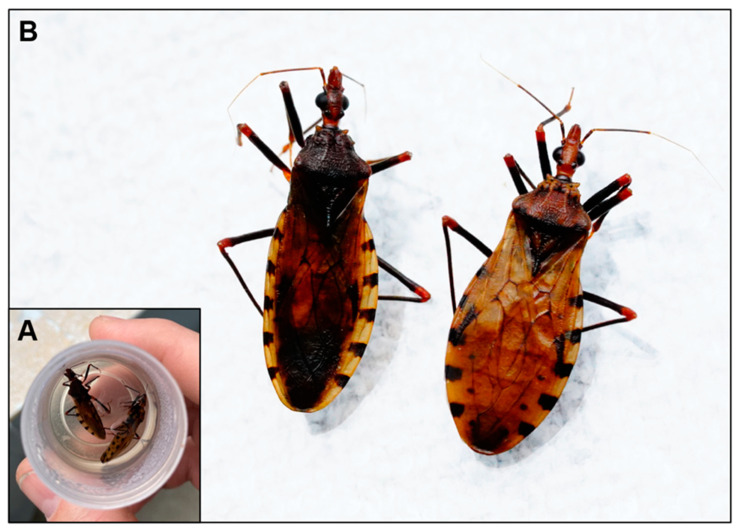
(**A**) Two adult triatomines were found inside a home in the city of Liborina (Antioquia Department). (**B**) Both triatomines were identified as adult male *Panstrongylus geniculatus* using morphologic keys [101]. *Trypanosoma cruzi* DTU TcI was detected in one of the triatomines via RT-PCR molecular techniques [97].

Peridomestic human exposure to triatomine vectors has been increasing in certain regions of Colombia and throughout other regions of Latin America and the United States [9,94,95,96,97]. Invasion from adult insects has been associated with confirmed or suspected cases of oral Chagas disease [5,14]. *P. geniculatus* (Figure 3) and *R. pallescens* are the triatomine vectors in Colombia that have been implicated in oral outbreak occurrences [9,14,20,25,98,99]. During warm weather, both species tend to invade human dwellings. Moreover, recent findings have revealed that both species carry high loads of *T. cruzi* in their hindguts [97,100]. These simultaneous biological characteristics could elucidate the significance of these species in facilitating oral transmission in Colombia.

Peridomestic triatomines are typically not well adapted to domiciliating the human dwelling, but during adult dispersal periods, the insects take flight in search of a mate and/or a blood meal. Ultimately, this can lead to being attracted to a neighboring human habitation. With anthropogenic changes and land development for humans and agriculture, we are now living and working closer to these natural landscapes where sylvatic triatomines reside. This allows for increased close contact with these triatomines that would normally not have exposure to humans or their companion animals.

**Figure 4:**
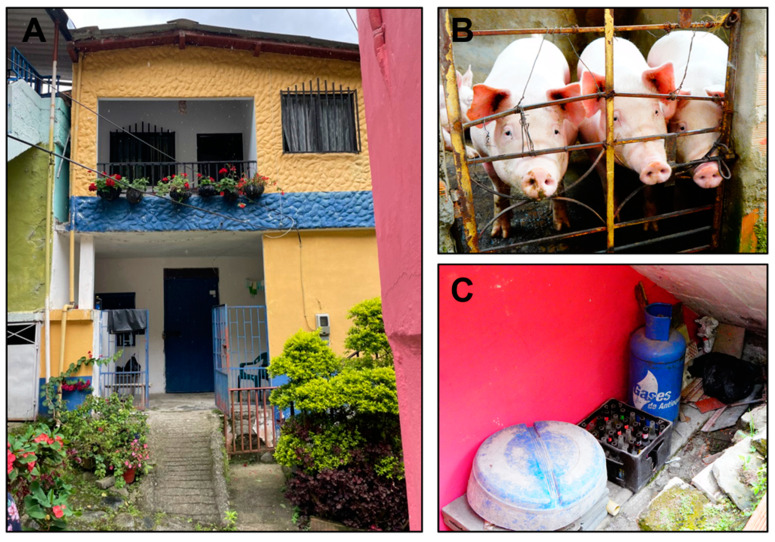
(**A**) Home in Liborina, Antioquia, Colombia, where several adult *Panstrongylus geniculatus* triatomines were found by homeowner inside on the second floor. The homeowners commonly left their windows open with no screens, and (**B**) domesticated pigs were housed below the structure. (**C**) Debris was located adjacent to the building, where evidence of small rodents could be seen.

Ecological and environmental factors associated with increased risk of peridomestic triatomine invasion in Colombia include housing structure(s) within or on the perimeter of the natural wild landscape, domesticated livestock near the home (pigs, chickens, cattle, horses, goats, and sighting of synanthropic mammals), outside lighting with fluorescent bulbs, stone piles, companion animals such as canines or felines living inside or outside inhabitants, lack of screens on windows and doors, home built above ground on stilts and wooden floorboards, debris collected near home serving as a habitat for small rodents and mesomammals such as opossums, and outdoor living areas where food and drink is prepared for consumption [102,103]. Other species, such as *T. dimidiata*, *T. venosa*, and *P. rufotuberculatus*, have also been shown to invade human dwellings in regions of Colombia where vectorial transmission is limited, but increasingly recognized cases of oral transmission have been documented. Vector control strategies for peridomestic triatomines are challenging because traditional methods of residual insecticide spraying can be evaded among flying insects into the home. An example of peridomestic invasion by *P. geniculatus* can be seen in a city-dwelling home in Liborina, Antioquia, Colombia, where a homeowner discovered adult insects inside on the second floor and alerted our research team. Further investigation found windows without screens, domesticated pigs adjacent to the structure, and some debris where small rodent droppings could be found under the stairwell (Figure 4). This scenario may lead to an increased attraction to a human dwelling and the ability for *P. geniculatus* to invade a home, but more research is needed to better understand the incidence of oral Chagas disease among those with peridomestic invasion of triatomines vectors in Colombia.

## 6. Opossums and Odoriferous Gland *T. cruzi*-Infected Secretions

**Figure 5:**
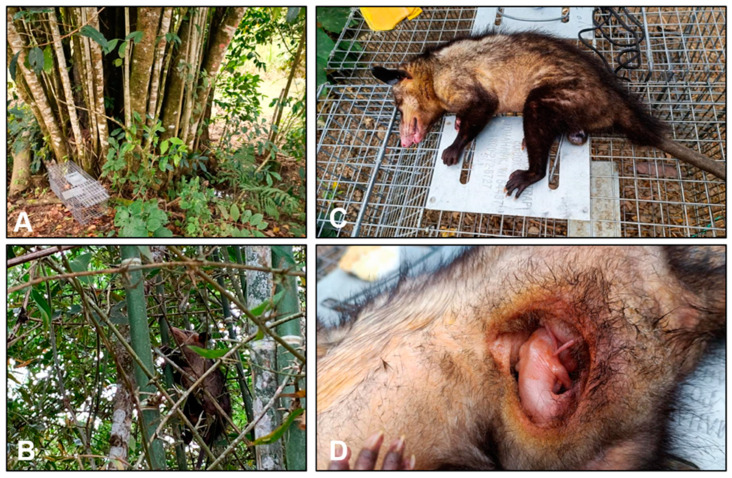
(**A**) Field trapping for *Didelphis marsupialis* in Miraflores, Boyacá Department, Colombia, near human dwellings and where suspected oral Chagas disease cases have been isolated. (**B**) *Didelphis marsupialis* found resting within canopy in Miraflores. (**C**) Female *Didelphis marsupialis* captured and anesthetized for *T. cruzi* infection testing. (**D**) Joeys were present in the marsupium, and concerns for vertical transmission of *T. cruzi* among *Didelphis marsupialis* are being investigated.

The significance of *Didelphis* species as an indicator of environmental disruption and its role as a primary reservoir of *T. cruzi* in human-impacted ecosystems has been described in Colombia and other regions of continental Latin America [104,105,106]. *Didelphis marsupialis* is a synanthropic mesomammal that plays an important dual role in both sylvatic and peridomestic *T. cruzi* transmission cycles. Domestic transmission is also postulated, and *D. marsupialis* has been found infected with *T. cruzi* near populated metropolitan regions [107]. Multiple studies have shown that *Didelphis* species harbor *T. cruzi* and infectious trypomastigotes [108,109,110]. In Colombia, *T. cruzi* DTU TcI has been detected from anal gland fluid collected among trapped opossums in regions where active vector-borne and oral outbreaks have been described, most notably in the eastern plains and Caribbean regions (Figure 5) [14,110]. Employing molecular tools such as next-generation sequencing (NGS) of *T. cruzi* isolates collected from humans and *D. marsupialis* in Colombia have demonstrated genetic similarities that point toward anal gland secretions as a source of human infection [111]. A recently described investigation of a suspected oral outbreak of CD in Cubara, Boyacá, Colombia, found a possible epidemiological linkage between humans and *D. marsupialis*. Among the five index human cases, all had *T. cruzi* DTU TcI detected in the blood, and subsequently, the same was found among the five *D. marsupialis* captured near the human dwelling. Only one triatomine (*P. geniculatus*) was found, and it was negative for *T. cruzi* [35]. The epidemiological importance of *Didelphis* species as a potential zoonotic source of oral transmission via anal gland secretions needs rigorous investigation. However, evidence suggests this is likely another potential source of oral transmission of *T. cruzi* to susceptible hosts, such as humans and companion animals.

## 7. Conclusions

A multitude of oral transmission routes can lead to the ingestion of *T. cruzi* in Colombia and likely other endemic regions of Latin America. More awareness is needed in endemic regions such as Colombia to help mitigate this form of transmission—particularly in areas where triatomine species are encroaching into new territories that culturally practice unpasteurized drink consumption. Governing health organizations, such as the Pan American Health Organization, should prioritize public health surveillance in these foci areas due to the high rate of severe disease associated with this infection route. Oral CD typically manifests with acute systemic symptoms, leading to severe illness and possibly death. This is largely due to the potential for significant high parasite loads during ingestion as opposed to other known routes, such as vector-borne. More research is needed to explain the pathogenesis of oral CD and why clinical manifestations are more robust and carry higher mortality. Anthropogenic landscape changes, increased exposure to infected peridomestic triatomines, ingestion of *T. cruzi*-contaminated food and drinks, and cultural practices such as consumption of wild bushmeat or raw armadillo blood have been shown to cause oral CD in Colombia. Ongoing investigations are showing that the anal gland secretions of the opossum (*Didelphis marsupialis*) can harbor infectious metacyclic trypomastigotes. This could potentially lead to zoonotic transmission of *T. cruzi* to humans and other susceptible hosts via contamination of the environment with odiferous secretions containing infectious parasites. More research is needed to better understand the life cycle of *T. cruzi*, the impact of understudied parasite DTUs and alternative popular food/drink products, and how contamination of the parasite among our food and drink products as well as the environment can lead to oral CD in Colombia.

## Figures and Tables

**Table 1:** Reported oral Chagas disease cases in Colombia from 1992 to 2023.

City/Department	Suspected or Confirmed Oral Source of *T. cruzi*	Number of Cases	Year	Reference
Tibú, Norte de Santander	Unknown	14	1992	Bohorquez, et al., 1992 [19]
Guamal, Magdalena	Palm wine	13	1999	Cáceres, et al., 1999 [20]
Lebrija, Santander	Orange juice	10	2008	Hernández, et al., 2009 [21]
Girón, Santander	Unknown	5	2008–2009	Zambrano, et al., 2010 [22]
Piedecuesta, Santander	Unknown	5	2008–2009	Zambrano, et al., 2010 [22]
Bucaramanga	Orange and/or tangerine juice	9	2009	Rameríz, et al., 2013 [23]
San Vicente de Chucuri, Santander	Triatomine fecal-contaminated food	3	2010	Rueda, et al., 2014 [24]
Aguachica, Cesar	Unknown	12	2010	Soto, et al., 2014 [25]
Turbo, Antioquia	*D. marsupialis* fecal and/or anal secretion contaminated food	11	2010	Ríos, et al., 2011 [26]
Paz de Ariporo, Casanare	Food/beverages contaminated by triatomine feces or *D. marsupialis* fecal and/or anal secretion	40	2014	Zuleta-Dueñas, et al., 2017 [27]
Trinidad, Casanare	Contaminated food	6	2015	Prensa Libre Casanare, 2015 [28]
San Luis de Palenque, Casanare	Contaminated food	4	2016	Instituto Nacional de Salud, BES, Número 18, 2017 [29]
Paz de Ariporo, Casanare	Contaminated food	4	2017	Prensa Libre Casanare, 2017 [30]
Puerto Colombia, Atlántico	Contaminated food	Family; 2 deaths	2019	Instituto Nacional de Salud, BES, Semena 33, 2019 [31]
Los Roble, Cesar	Contaminated food	3	2019	Instituto Nacional de Salud, BES, Semena 33, 2019 [31]
Maní, Casanare	Contaminated food and/or beverages	22	2019	Instituto Nacional de Salud, BES, Semena 33, 2019 [31]
San Luis, Antioquia	Contaminated food	4	2019	Instituto Nacional de Salud, BES, Semena 33, 2019 [31]
San José del Palmar, Chocó	Armadillo blood	2	2019	Instituto Nacional de Salud, BES, Semena 33, 2019 [31]
Yopal, Casanare	Contaminated food	4	2020	Prensa Libre Casanare, 2020 [32]
La Jagua de Ibirico, Cesar	Contaminated food	11	2021	Instituto Nacional de Salud, BES, Semana 20, 2021 [33]
Trinidad, Casanare	Contaminated food	1	2021	Instituto Nacional de Salud, BES, Semana 6, 2021 [34]
Cubara, Boyacá	*D. marsupialis* fecal and/or anal secretion contaminated food	5	2021	Gutiérrez, S. A., et al., 2023 [35]
Sardinata, Norte de Santander	Contaminated food	3	2022	Instituto Nacional de Salud, BES, Semana 11, 2022 [36]
Arauquita, Arauca	Contaminated food	3	2022	Instituto Nacional de Salud, BES, Semana 18, 2022 [37]
Maracavita, Santander	Contaminated food	3	2022	Instituto Nacional de Salud, BES, Semana 18, 2022 [37]
Becerril, Cesar	Contaminated food	11	2022	Instituto Nacional de Salud, BES, Semana 18, 2022 [37]
Tauramena, Casanare	Contaminated food	1	2023	Instituto Nacional de Salud, BES, Semana 14, 2023 [38]
Granada, Meta	Contaminated food	40	2023	Instituto Nacional de Salud, BES, Semana 39, 2023 [39]

## Data Availability

Not applicable.
